# Generation of a network slicing dataset: The foundations for AI-based B5G resource management

**DOI:** 10.1016/j.dib.2024.110738

**Published:** 2024-07-15

**Authors:** Miquel Farreras, Jordi Paillissé, Lluís Fàbrega, Pere Vilà

**Affiliations:** a Institute of Informatics and Applications, Universitat de Girona, C/ de la Universitat de Girona, 6, 17003 Girona, Spain; b UPC-BarcelonaTech, Carrer de Jordi Girona, 31, 08034 Barcelona, Spain

**Keywords:** 5G, B5G, Deep Learning, Network simulation, Network slicing, Quality of service, Transport networks, KPI

## Abstract

This paper presents a comprehensive network slicing dataset designed to empower artificial intelligence (AI), and data-based performance prediction applications, in 5G and beyond (B5G) networks. The dataset, generated through a packet-level simulator, captures the complexities of network slicing considering the three main network slice types defined by 3GPP: Enhanced Mobile Broadband (eMBB), Ultra-Reliable Low Latency Communications (URLLC), and Massive Internet of Things (mIoT). It includes a wide range of network scenarios with varying topologies, slice instances, and traffic flows. The included scenarios consist of transport networks, excluding the Radio Access Network (RAN) infrastructure. Each sample consists of pairs of a network scenario and the associated performance metrics: the network configuration includes network topology, traffic characteristics, routing configurations, while the performance metrics are the delay, jitter, and loss for each flow. The dataset is generated with a custom network slicing admission control module, enabling the simulation of scenarios in multiple situations of over and underprovisioning. This network slicing dataset is a valuable asset for the research community, unlocking opportunities for innovations in 5G and B5G networks.

Specifications Table

**Table:** 

Subject	Computer Science/Computer Networks and Communications
Specific subject area	5G and B5G, network slicing, network performance (KPI)
Type of data	Network performance data from packet-level simulations
Data collection	Using a custom network simulator, BNNetSimulator, based on OMNeT++, multiple topologies, traffic and routing configurations are used to simulate a wide range of network slicing scenarios. Under and overprovisioning are tested, with a custom access control written in Python to avoid exceeding the capacity of the links. The topologies are obtained from the Internet Topology Zoo [2], while the routing configurations are specified using the NetworkX Python library [3], and randomly chosen to emulate a more realistic scenario. The results of each simulation are saved in .txt files, and compressed in a unique .tar.gz file for each simulation.
Data source location	Universitat de Girona/ UPC-BarcelonaTech
Data accessibility	Repository name: Generation of a network slicing dataset: the foundations for AI-based B5G resource management Data identification number: 10.5281/zenodo.10610616 Direct URL to data: https://zenodo.org/records/10610616

## 1 Value of the Data

• Currently there is a lack of datasets dedicated to network slicing suitable for training machine learning models. Our research aims to address this gap by generating new datasets using a network simulator. Specifically, our goal is to facilitate the training of models to predict network Key Performance Indicators (KPIs). An initial simplified scenario is developed to check the correctness of the simulations, followed by extensive simulations of various topologies to improve model generalization and evaluation.
• This network slicing dataset, generated through a realistic packet-level simulator, BNNetSimulator, encompasses diverse scenarios, including different slice instances, traffic flows and Quality of Service (QoS) requirements for eMBB (enhanced Mobile BroadBand), URLLC (Ultra-Reliable Low Latency Communications), and mIoT (massive Internet of Things) applications. It facilitates rigorous evaluation of AI/ML models and algorithms for network performance prediction and flow allocation, especially in Service Level Agreement (SLA)-constrained scenarios.
• Researchers in 5G and B5G networks, Artificial Intelligence, and network slicing can leverage this dataset for in-depth analysis, model training, and performance evaluation, especially regarding resource allocation algorithms. Network architects and engineers can use the dataset to consider diverse scenarios, aiding in the design and optimization of slicing strategies for different applications.
• Other researchers can reuse this data to advance their work, validating and comparing existing models or developing new ones for predicting network metrics such as delay, jitter, and loss. The dataset also offers opportunities for experimentation with different network configurations, traffic scenarios, and slicing strategies.
• This dataset serves as a benchmark for comparing different algorithms for network KPI prediction, serving as a starting point to develop and test new and improved traffic models.

## 2 Background

The motivation behind creating this dataset stems from the lack of comprehensive datasets for network performance data, particularly in the context of network slicing for training machine learning models. To address this gap, our research aims to generate new datasets using the BNNetSimulator network simulator.

## 3 Data Description

The dataset comprises pairs of (*scenario, performance*). The *scenario* includes all configuration parameters of the network scenario, such as the number and type of slices, traffic flows in each slice, packet arrival processes, Weighted Fair Queuing (WFQ) weights, topology, and routing configurations. The *performance* includes the KPIs needed to validate if the SLAs of a slice are met, such as per-flow delay, jitter and packet loss.

The dataset consists of a set of 7900 simulation results. There are three folders with 7900 files each (one with network topologies, one with routing configurations and another with slices). In addition, 79 .tar.gz compressed files are included. Each compressed file has 6 files with 100 results inside. Each line of each file represents the results of a unique simulation.

**Fig. 1:**
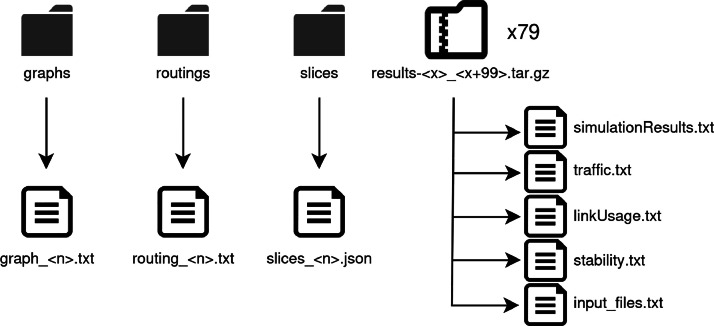
Folders and files structure of the dataset.

Each simulation is composed by: one file with network topology data (*graph_n.txt*), one file with routing configuration data (*routing_n.txt*) a file with slice data (slices_n.json), and the corresponding line inside the compressed .tar.gz file containing n in the range. The folders and files structures are represented in Fig. 1. The x in the .tar.gz names is between [0,7800]

Firstly, network topologies used for each simulation are saved in the *graphs* folder. This file contains the network topology in GraphML format [4], with nodes, queues and edges. Each network topology is chosen randomly from the Internet Topology Zoo [2], and the required additional attributes are added to each file. One global attribute is levelsToS, which indicates the total number of flows in the network. The node attributes are:

- id: node unique identifier in the simulation.
- type: the values can be “transport”, “antenna”, “destination_embb”, “destination_mmtc” or, “destination_urllc”. It represents the node role. Antenna nodes generate traffic, transport nodes forward traffic, and destination nodes receive traffic of a single type of slice.
- schedulingPolicy: “FIFO” (First-In First-Out) or “WFQ” (Weighted Fair Queuing)
- schedulingWeights: List of weights separated by commas associated to each QoS queue. Used only when schedulingPolicy is “WFQ”.
- levelsQoS: Number of supported QoS classes in the queues
- queueSizes: queue capacities (in number of packets) for each output queue of the node. Values are separated by commas. Each value is associated with a QoS queue.
- tosToQoSqueue: list separated by commas, containing the types of service assigned to each queue. If levelsQoS is larger than 1, each list of tosToQoSqueues is separated by a semicolon.

The attribute tosToQoSqueue is used with the Weighted Fair Queues (WFQ) to identify each flow, and then map them to the corresponding queue during the simulations.

Secondly, the routing configurations are saved in the *routings* folder. Each file is a .txt file containing the routing matrix (R) for each node. The filename, using the same format as the network topology files, is *routing_n.txt*, where *n* is the simulation id. It can be read as a CSV file, where each cell R[source][destination] in the routing matrix contains the port in the node used to follow the path to the destination.

Thirdly, the network slices are saved in the *slices* folder. These files are formatted in JSON [5], containing the network slices assigned in each simulation. The filename is *slices_n.json*, where *n* is the simulation id. Each file contains a list of slices. Each slice contains a list of flows. Note that one simulation can contain multiple instances of the same type of slice, and that each slice contains multiple flows. The slices attributes are:

- Delta (δ): value within the range [0,1], that determines whether the reserved average bit rate of a flow is close to the minimum flow bit rate or close to the maximum flow bit rate.
- Type: usage type of the slice. We consider the three slice types defined by 3GPP [10]: Enhanced Mobile Broadband (eMBB), massive Internet of Things (mIoT), and Ultra Reliable Low Latency Communications (URLLC).
- Number: numeric identifier for the slice. Each slice type has its identifiers. Slice instances can be uniquely identified by the slice type and number.

Each flow has a unique origin and a destination node. The flows attributes are:

- Origin: node where the traffic flow originates. It represents a device connected to an antenna node.
- Destination: node where the traffic flow is received.
- Origin_node_antenna: node where the origin_node is connected. It represents the antenna node.
- Path: list of hops of a flow, including origin and destination nodes.
- Bandwidth: integer representing the bits per second of the traffic generated.
- Traffic_string: traffic properties used in the *traffic.txt* inside the .tar.gz file, including:
  - Type of traffic (Poisson in this case, represented as 0)
  - Bandwidth generated by the flow in bits per time unit, average number of packets per time unit, and exponential maximum factor (used for the Poisson distribution).
  - Packet size distribution: size distribution, average packet size and minimum/maximum packet sizes only for binomial distribution.

Finally, the results of each simulation are saved in a .tar.gz file. Each .tar.gz file contains groups of 100 simulations to reduce the total size and number of compressed files. Each .tar.gz contains .txt files, which contain 100 lines each. Each common line in each file represents an individual simulation. The files are briefly described below:

- simulationResults.txt: it contains all the flow and link information necessary, such as delays, jitter and losses. More details on the data represented in this file are included in the subsection DataNet API features.
- traffic.txt: input file used for defining the traffic of each simulation. It contains the same format strings concatenated as in Traffic_string for each flow.
- linkUsage.txt: it includes the occupancy of each queue of each output port of all links.
- stability.txt: it contains the iteration time, curly stability, status at the end, memory used and time in seconds elapsed.
- input_files.txt: This file indicates which network topologies, routing configurations and slices correspond to each line of the previously described files.

### 3.1 DataNet API features

**Fig. 2:**
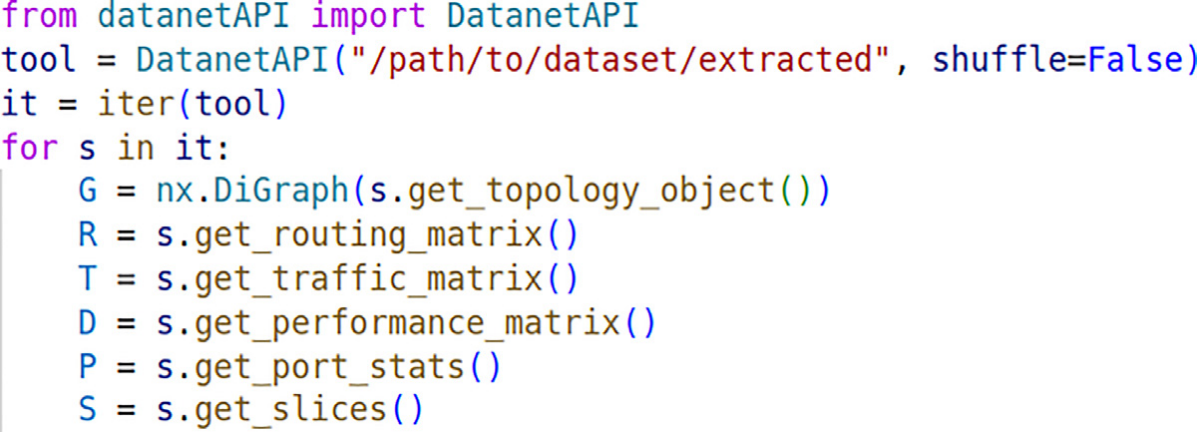
basic code to iterate through each sample of the dataset.

To facilitate reading the dataset contents for each simulation, the datanetAPI.py Python file is provided in the repository. It is based on the DataNetAPI project [6], but extended to also process the network slicing data collected in this work. To use it, it can be called from a Python code as shown in Fig. 2. Then, creating an iterator for the dataset and obtaining the data structures.

The shuffle option can mix the different samples (e.g. for providing an input to a training Machine Learning algorithm).

Each sample contains the following features:

- global_packets: total number of packets transmitted in the network per time unit (packets/time unit).
- global_losses: total number of packets lost in the network per time unit (packets/time unit).
- global_delay: Average per-packet delay over all the packets transmitted in the network (time units).
- maxAvgLambda: overall traffic intensity of the network scenario. Maximum average traffic rate (bits/time unit) that a path can generate in the simulation scenario. Note that this traffic rate may be split into several flows, sending traffic over the same src-dst path.
- performance_matrix: Matrix with aggregate source-destination and also flow-level performance measurements (delay, jitter and loss) measured on each source-destination pair.
- traffic_matrix: Matrix with the time and size distributions used to generate traffic for each source-destination pair.
- routing_matrix: Matrix with the paths to connect every source-destination pair.
- topology_object: NetworkX graph object including topology-related information at the node and link-level.
- links_performance: list of dictionaries with the performance metrics associated with each link.
- port_stats: list of dictionaries with the performance metrics associated with each output port.

Detailed descriptions can be found for each data structure in the file *features.pdf* in [1].

## 4 Experimental Design, Materials and Methods

Data is obtained through simulation using thea packet-level network simulator [7,12], BNNetSimulator, based on OMNeT++ 5.6.2 discrete event simulation framework [8]. This simulator is modified to accommodate different weights for each Weighted Fair Queuing (WFQ) port, as the original code does not support this feature. This change, coupled with the addition of the new input files that include network slicing properties, and the adaptation of the DataNetAPI to use these files, enables the simulator to generate new network slicing simulations.

**Fig. 3:**
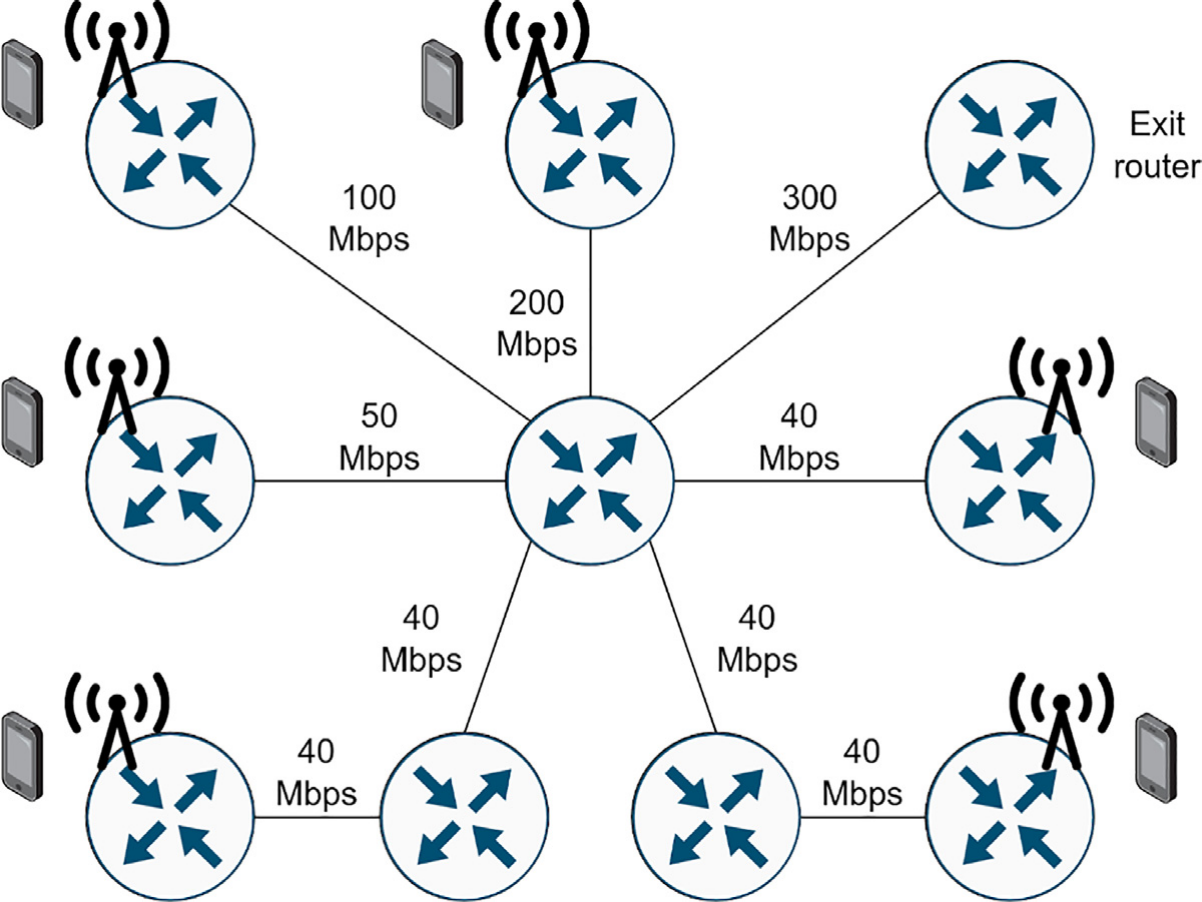
An example of a network topology: routers with antennas (the RAN part) would be the source nodes of the transport network while the exit router at the other end would be the destination node.

Multiple network topologies, traffic matrices, routing configurations and slice allocations are considered. Traffic flows originates from different routers connected just after the Radio Access Network (RAN) part and exits the network to a router at the other end (Fig. 3 shows a simplified network example). The users are connected to the RAN using their User Equipment (UE). Each user is represented as a node connected to its origin router, represented with an antenna. That flow is then routed to the exit router shown in the right. A flow can represent either a single UE, or a device that uses different network slices, i.e. it sends data with different connections that have different QoS requirements. Therefore, a slice consists of a set of traffic flows from different sources to destinations with a common set of performance requirements. The allocation of network resources to slices is done in the following way:

- The output links of nodes use the Weighted Fair Queuing (WFQ) queueing discipline. Traffic is classified in several “virtual” WFQ queues, and each queue is assigned a weight that determines the minimum portion of the link capacity that the traffic going through that queue will receive. WFQ provides “isolation” among queues in the sense that excess traffic in one queue does not affect the traffic in other queues, i.e., each queue has a guaranteed minimum portion of the link's capacity.
- Each WFQ queue is assigned to an individual, specific slice, and the flows of this slice share the same queue. The size of each WFQ is static for all queues in each simulation, 32 packets.
- Each flow has a reserved bit rate, Vreserved, and their sum over all flows of a slice determines the required portion of the link capacity and therefore the queue weight; the sum of the reservations never exceeds the link's capacity.
- Given a flow with an average bit rate, its Vreserved lies between two values, the minimum bit rate Vmin(below the average) and the maximum bit rate Vmax (above the average); a Vreserved close to Vmin represents a situation of resource underprovisioning and close to Vmax of overprovisioning. The weight of the slice in each WFQ queue is calculated using the Vreserved, assigning the weight as the percentage of the total link capacity that the Vreserved uses.

The network scenarios simulated in this dataset aim to reflect real-world conditions by incorporating diverse topologies, realistic traffic patterns, and various network slicing configurations. The use of the Internet Topology Zoo ensures that the topologies are based on actual network structures. Moreover, the traffic patterns and slice configurations are designed to mimic real-world applications, such as eMBB for media delivery, URLLC for high-reliability applications, and mIoT for IoT devices. Slices exhibit the following characteristics:

- Three different network slices types (eMBB, mIoT, URLLC) with a variable number of instances.
- The proportion of slice types is variable in each simulation, with each slice possibly existing in one or multiple antennas.
- Each slice has at least one assigned flow.
- At least one eMBB flow is included for each simulated antenna, serving as the standard network usage. The remaining flows for each access antenna and slice are randomly assigned.
- Flows are created to fill link capacities, and reservations are made based on each flow's required average bit rate Vreserved.
- The bit rate reservation for each slice is allocated on each output port using WFQs. This allows the implementation of resource isolation of each slice from the others, a key characteristic for network slices.
- Each slice instance contains different flows with the traffic characteristics outlined in Table 1.

**Table 1:** Slice types considered and flow characteristics [9,11].

Flow type	Maximum delay	Maximum packet loss	Intended use	Bit rate	Packet size
eMBB	10 ms	10 %	Broadcasting, media delivery, general usage	18 Mbps	Average: 6000 bits Minimum: 12000 bits Maximum: 18000 bits
URLLC	0.5 ms	0.001 %	High reliability, ultra-low latency, high availability	3.5 Mbps	Average: 800 bits Minimum: 256 bits Maximum: 1600 bits
mIoT	Up to 10s	1 %	Long battery, low cost devices, extreme coverage	0.2 Mbps	Average: 160 bits Minimum: 320 bits Maximum: 480 bits
- A standard proportion of capacity of 1 eMBB flow, 7 URLLC flows and 2 mIoT flows is applied. These proportions are only used for defining the random probability of assigning a flow to an antenna node, not being used later for the flow creation.

Simulations allow different scenarios, ranging from underprovisioning to overprovisioning, controlled by a tunable parameter δ in [0,1] that adjusts the amount of under and overprovisioning of the *V_reserved_* as shown in the following equation,

$$V_{reserved}=V_{min}+\delta \left(V_{max}-V_{min}\right)$$

where *V_reserved_* represents the actual reserved bit rate for the specific flow, δ is the under/ overprovisioning parameter, with a random value between [0,1] for flows in the same slice, and *V_min_* and *V_max_* are the minimum and maximum bit rates, respectively. This allows for a range of reserved bit rates from an underprovisioning of *V_min_* to an overprovisioning of *V_max_*. The maximum and minimum bit rates are calculated as the 120 % and 90% of the average flow bit rate for the overprovisioning and underprovision situations, respectively.

To facilitate the simulations, each source of a flow is technically a source node added to the network, connected to the antenna where it is located. The destination nodes of the generated flows are common for each type of flow, located at the right port of the exit router in the example network topology (Fig. 3).

To sum up, the steps to generate each simulation are:

(1) Randomly select a network topology from Internet Topology Zoo [2].
(2) Randomly assign link capacities from the predefined range, between 25 and 300 Mbps.
(3) Designate at least two nodes as antennas. A maximum of N/3 nodes will be source nodes, where N is the number of nodes in the network topology.
(4) Define the three destination nodes (eMBB, mIoT, URLLC). The remaining nodes are defined as transport nodes.
(5) Set the number of slice instances randomly between 1 and N.
(6) Specify the slice type and traffic for each slice.
(7) Assign one eMBB flow for each source, while the others are determined by the capacity and reservations of the network. Routing is achieved using the NetworkX Python library [3], obtaining firstly the shortest path and using the other possible routes when a path is completely provisioned.
(8) Generate flows assigned to the different slices, controlling that the sum of the reservations for each link does not exceed the capacity of the link.
(9) Remove empty slices.
(10) Execute the simulation on the network simulator.

In terms of individual samples, the node count ranges from 16 to 764 nodes. The simulations stop when the stationary state is detected, where the state of the simulation reaches the equilibrium level at a certain point. That is, when the difference between the maximum and the minimum of the delays, divided by the mean delay, is smaller than 0.001 during two evaluation windows. Simulation time spans between 1.4 hours and 30.4 hours, while the memory utilization varies from 217 MB to 30 GB.

The hardware used to run the simulations was a cluster with 40 computing nodes, each with two Intel Xeon E5-2630L v2 2.40 GHz CPUs, 128 GB of RAM and 1 TB disks. The simulations were allocated in the cluster (sharing resources with other processes), with a limitation of 30 GB of RAM.

Some metrics of the simulations are shown in Table 2. It can be observed that, as expected from the characteristics of the types of slice, the average number of eMBB slices is lower due to the higher bit rate of each slice, while there are many more mIoT slices due to their lower bit rate.

**Table 2:** metrics of the generated simulations.

Metric	Value
Total samples	7900
Number of different topologies	130
Average samples per topology	61
Average total number of slices per sample	219
Average total number of eMBB slices per sample	9.2
Average total number of URLLC slices per sample	45.6
Average total number of mIoT slices per sample	164.9

**Fig. 4:**
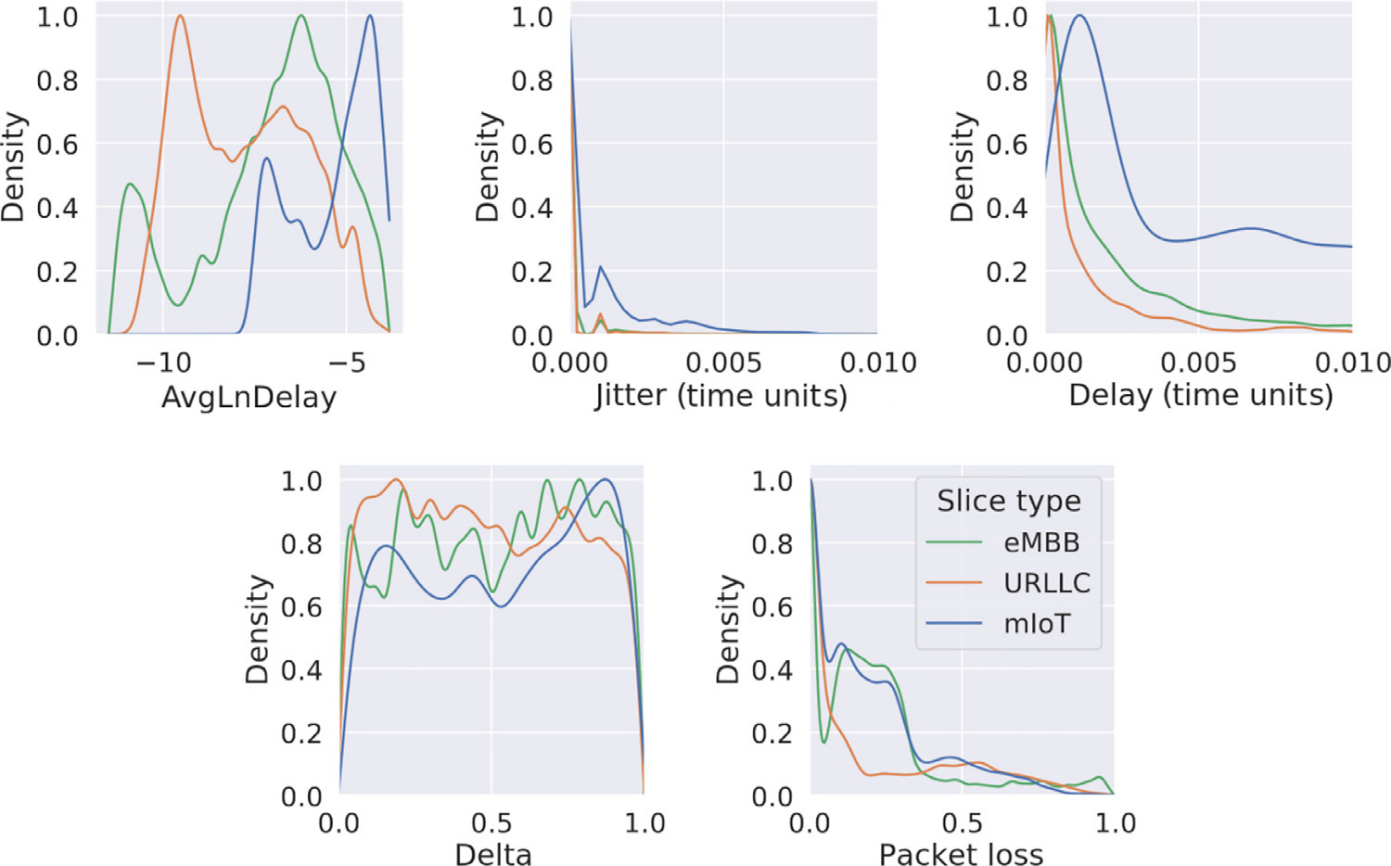
Probability Density Function (PDF) of several flow features for each slice type.

We have analyzed the distribution of the values for the different set of features at the flow, queue, and link level. Fig. 4 shows the Probability Density Function (PDF) of values of the numeric features of the slices. For each type of slice, it can be observed that the delay and logarithm of the delay (AvgLnDelay) is higher for the mIoT slices, while it is lower for the URLLC and eMBB slices. The jitter is low in all cases, being slightly higher for the mIoT slices. The jitter is calculated as the standard deviation of the variance of the delay. The delta (δ) values are mostly uniform for each type of slice, while the packet loss is lower in the URLLC slices. We can see that the parameters present a wide range of values in order to cover as many scenarios as possible. For example, the delta parameter presents a quasi-uniform distribution between 0 and 1.

**Fig. 5:**
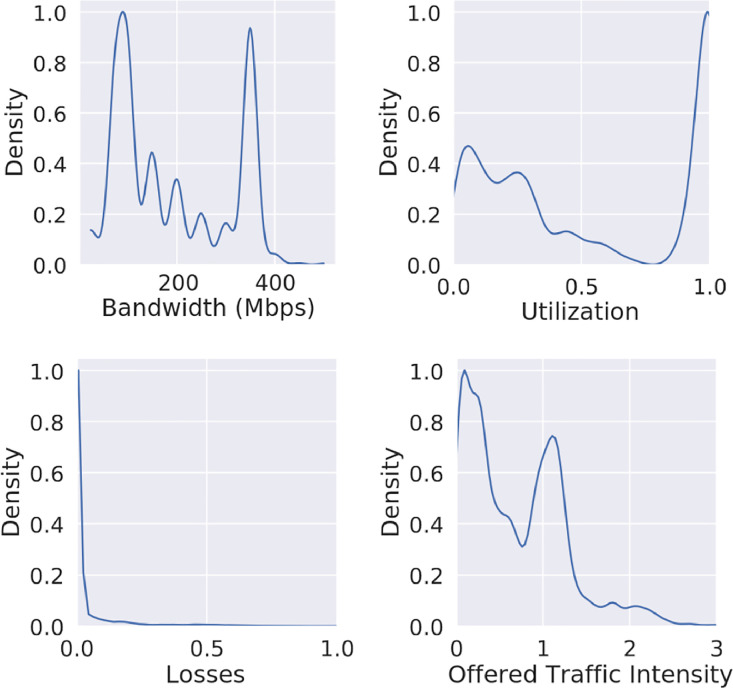
Probability Density Function (PDF) of several link features for each slice type.

Fig. 5 shows the Probability Density Function (PDF) of several link features for each slice type., showing high utilization of the links, as it was intended in the scenarios, low losses in most cases, various bandwidths and an offered traffic intensity (O_t_) with most values around 0 or 1, although other values have also been generated. O_t_ is a feature calculated from other existing features. It is defined as the sum of traffic (T_f_) of all flows f passing through the network link N_l_ divided by the bandwidth C_l_ of that link, resulting in a scalar feature assigned to the link state:

$$O_{t}=\frac{\sum_{f\in N_{\ell }T_{f}}}{C_{\ell }}$$

The links that use FIFO queues, which connect each source of a flow to the antenna node, are excluded from the analysis, as they are overdimensioned for any type of traffic and therefore are not significant for the WFQ slicing behavior.

**Fig. 6:**
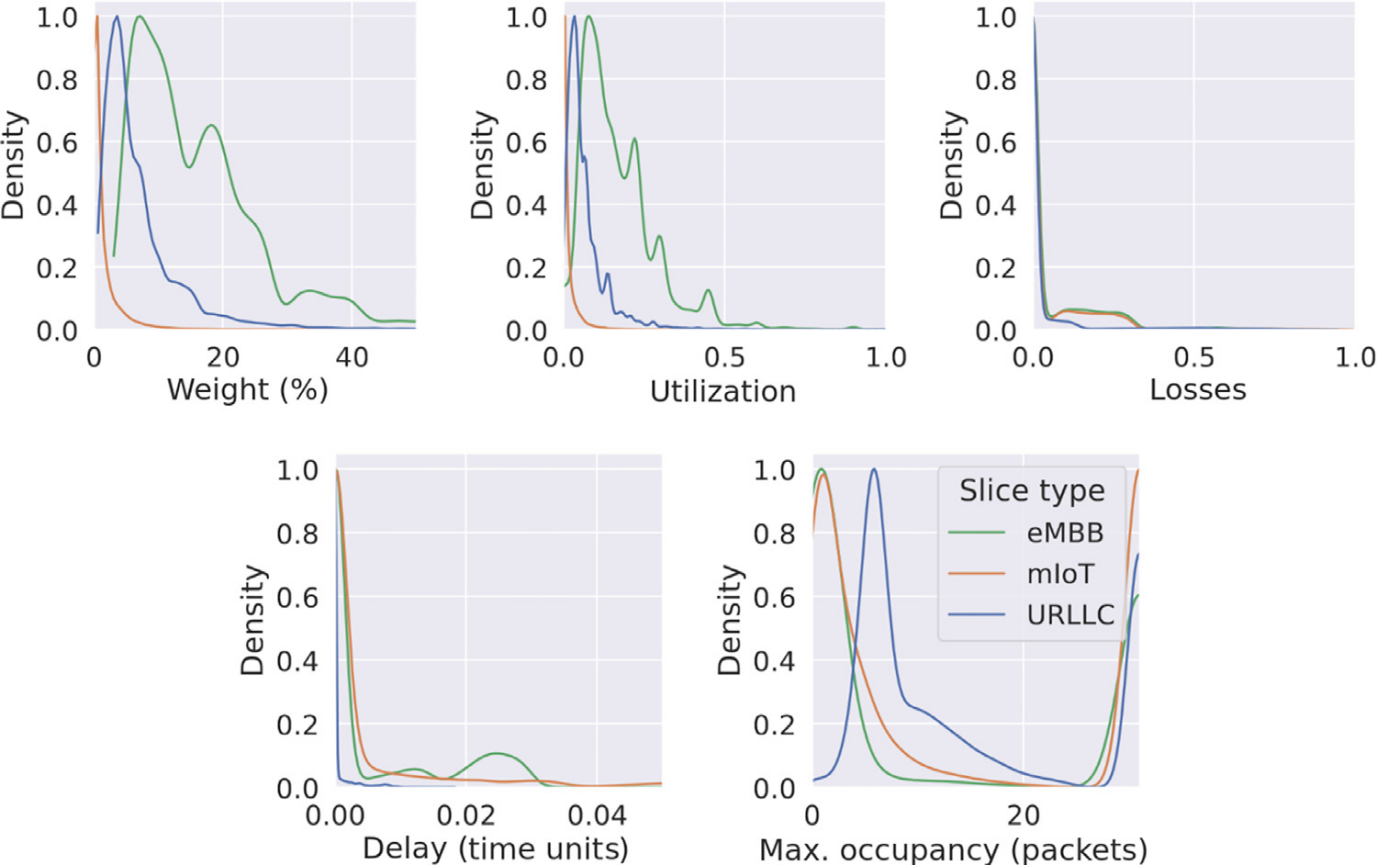
Probability Density Function (PDF) of several queue features for each slice type.

Fig. 6 shows the Probability Density Function (PDF) of several queue features for each slice type. It can be seen that most weights of the WFQs are under 50%. The utilization of the queues is higher for the eMBB slices, while it is very low for the mIoT case. This is due to the fact that eMBB slices are the ones with the most bandwidth assigned. The losses are lower in the URLLC queues analyzed as also seen in the links, while the delays are also lower in the URLLC. The maximum queue occupancies, represented in number of packets, are shown to be a little bit higher in the URLLC case on average, but the total maximum queue occupancy is reached more commonly by the mIoT slice types. We can see that the queues are full in some situations. This is expected, in order to have some packet loss.

The dataset includes 7,900 simulation results, each corresponding to a unique combination of network topology, routing configuration, and network slice allocation. This diversity is critical as it reflects different network configurations and complexities encountered in real-world scenarios. 129 different network topologies have been used, meaning that, on average, 61 different scenarios have been built on each network topology. This repetition allows for variations in traffic patterns, routing decisions, and network slice allocations to be explored. By varying these parameters, we can observe how each topology behaves under different conditions, encompassing a wide range of operating conditions. This can help the models trained using this dataset to capture the behavior of the network under multiple conditions.

## 5 Discussion

In order to validate the representativeness of our dataset, we have carried out statistical analyses to ensure that the performance metrics are not biased towards any specific topology or scenario. This includes checking the distribution of performance metrics across different topologies and scenarios to ensure a balanced representation. However, despite our efforts to create a realistic dataset, some simplifications and assumptions were required:

- Exclusion of Radio Access Network (RAN): The simulations focus on the transport network, excluding the RAN infrastructure. This allows for a detailed analysis of the transport network.
- Traffic patterns: The traffic generation is based on Poisson processes, which, while commonly used, may not fully capture the bursty nature of specific real-world traffic.
- Fixed routing configurations: Routing decisions in the simulations are fixed for each scenario and do not adapt dynamically to changing network conditions, which can occur in real-world networks. This is partially addressed by simulating multiple scenarios with different routings on each network topology.
- Fixed UE location: The users are static in each simulation. Nevertheless, the aim of this dataset is to obtain a picture of the performance of the network with a specific configuration. Therefore, if we perform a new simulation with the same network topology, but re-attaching some of the source traffic nodes to a different antenna node, we can obtain performance estimations about mobility events between different RAN sections.

## Limitations

Network topologies have link capacities of up to 500 Mbps and a maximum number of nodes of 764; going beyond would have required much more computational resources and simulation time, and these values achieved a good balance between simulating realistic scenarios and an affordable simulation time. Three types of slices have been considered, eMBB, URLLC and mIoT, and although there are others, these are the most common. The flow's traffic model for the all three types of slices has been the same and consisted of exponential inter arrival packet times and binomial packet size, but with different values for each slice type to model their different characteristics. Finally the network scenarios consider the transport network and the RAN part is not included. Future work could involve dynamic routing protocols, more traffic models and RAN components.

## Ethics Statement

We confirm compliance with ethical requirements for publication in Data in Brief. Our work does not involve human subjects, animal experiments, or data from social media platforms.

## CRediT authorship contribution statement

**Miquel Farreras:** Conceptualization, Methodology, Software, Writing – original draft, Writing – review & editing. **Jordi Paillissé:** Conceptualization, Methodology, Software, Writing – original draft, Writing – review & editing. **Lluís Fàbrega:** Conceptualization, Methodology, Software, Writing – original draft, Writing – review & editing, Supervision. **Pere Vilà:** Supervision, Conceptualization, Methodology, Software, Writing – original draft, Writing – review & editing.

## Data Availability

Generation of a network slicing dataset: the foundations for AI-based B5G resource management (Original data) (Zenodo).
